# Assesment of accessory branches of canalis sinuosus on CBCT images

**DOI:** 10.4317/medoral.23235

**Published:** 2019-12-24

**Authors:** Dilara Nil Tomrukçu, Taha Emre Köse

**Affiliations:** 1 Assistant Professor, Recep Tayyip Erdogan University, Faculty of Dentistry, Oral and Maxillofacial Radiology Department

## Abstract

**Background:**

The aim of this study is to describe the presence, to reveal the frequency and characteristics of accessory canals (ACs) of the canalis sinuosus (CS) by cone beam computed tomography (CBCT).

**Material and Methods:**

A total of 326 CBCT examinations were scanned retrospectively. The anatomical views were evaluated on sagittal, axial, coronal and cross sectional imaging. The following parameters were recorded: age, sex, presence or absence of ACs, location in relation to the adjacent teeth and distance to the nasal cavity floor (NCF), alveolar ridge crest (ARC) and buccal cortical bone (BCB), and incisive canal. All the collected data were statistically analyzed.

**Results:**

113 patients (34,7%); presented ACs in total 214 foramina of the sample. There were no statistically significant changes in the presence of ACs regarding age groups excluding 80-89 years. But there is a statistically significant difference regarding the frequency of ACs and the gender. The prevalence for male patients was higher than female patients. Curved-shape configuration of CS prevalence is found as 69,15%. The prevalence of vertical tracing is 26,16% and Y-shape configuration of CS prevalence is 4,67%. Diameter of the foramens of the CS branches was 1.30 mm. The mean distance of the AC to the NCF, BCB, and ARC were found 13,83 mm, 6,60 mm and 5,32 mm, respectively.

**Conclusions:**

In the anterior palatal region, ACs are mostly related to CS’s branches. So; knowing the course of CS branches in surgical planning and radiographic evaluations in this region is extremely important for preventing complications and avoiding misdiagnosis.

** Key words:**Anterior superior alveolar nerve, canalis sinuosus, maxilla.

## Introduction

Frequently, several surgical procedures are performed in the anterior maxillary region such as placement of dental implants, surgical removal of impacted or supernumerary teeth, periodontal surgery, endodontic surgery, orthognathic surgery and cyst therapy (1). The neurovascular supply of this region is caused by the maxillary nerve, which is separated from the trigeminal nerve, which is the fifth cranial nerve, and by the vessels accompanying this nerve. The infraorbital nerve separated from the maxillary nerve innervates the skin and the mid-face region. The anterior superior alveolar (ASA) nerve is a branch of infraorbital nerve and innervate incisors, canines, and soft tissues (2). Another important anatomical structure in the premaxillary is nasopalatinal canal. The vessels and nerves of the same name passing through the canal are distributed to the anterior teeth and soft tissues and supply this region (3). There are many accessory foramina in this region and these anatomical variations of various size and morphological features can be misdiagnosed and mixed with apical pathologies (4).

Canalis Sinuosus (CS) was one of the accessory canals in this region and was first described by Jones in 1939 (5). The canal was named due to its double-curved course (4). This small canal is originating from infraorbital canal, and medially bent to anterior wall of the maxillary sinus, passing below the infraorbital foramen. Reaching the anterior margin of the nasal aperture in front of the anterior end of the inferior concha, it follows the lower margin of the aperture and opens next to the nasal septum in front of the incisive canal. Within the CS, the arteries and veins of the same name are accompanied by the ASA nerve (5,6).

Panoramic and periapical radiographs used in routine are not sufficient to diagnose accessory channels due to some limitation (superimpositions, magnifications, distortions, low image quality). Thanks to the high resolution, cross sectional view and diagnostic reliability at lower costs and radiation doses advantages in dentistry, the use of CBCT has made it easier to diagnose these anatomical structures (7). Limited information about this anatomical structure, insufficient literature and conventional radiography cannot show the accessory structures properly, it may make their diagnosis difficult, may lead to misdiagnosis, and may cause complications during or after surgical procedures (4,8).

The aim of this study is to determine the location and diameter of accessory canals (ACs) and to correlate the canal with gender, age and distance from the major structures such as the buccal cortical bone (BCB), nasal cavity floor (NCF), alveolar ridge crest (ARC), and the incisive foramen.

## Material and Methods

This study was designed as a retrospective analysis of CBCT images obtained between January 2017 and May 2019 in the Department of Oral and Maxillofacial Radiology, Recep Tayyip Erdogan University, Faculty of Dentistry. All patient identifiers were removed from the image files. This research was approved by the Ethics Committee of the Recep Tayyip Erdogan University Faculty of Medicine with the number 2019/28. Patients who showed the following criterias were excluded: those who had undergone surgical procedures or bone grafting in the anterior maxilla; those presenting pathological lesions or trauma (plate and screws) in the anterior maxilla; and those whose CT scans failed to present satisfactory quality. Only the ACs with foramina in the maxilla included in the study. CBCT images of 326 patients were screened. The following parameters were registered: sex, age, number of ACs, diameter of ACs, location of the AC in relation to the adjacent teeth. Distance from NCF, ARC and BCB distances was measured via Manhães Júnior *et al*’s method (9). In addition, the types of the ACs categorized via Von Arx *et al* (1) as curved, vertical and y shape. And the diameter of each canal was measured at the median distance of its total length at axial slices. Axial, coronal and sagittal slices with 0,2 mm interslice distance and 0,2 mm slice width were analyzed in every case. Diameters were determined by measuring the palatine opening of the AC on both coronal and cross-sectional images. All CBCT images were obtained with Planmeca ProMax 3D Classic (Planmeca Promax 3D; Planmeca Oy; Helsinki, Finland) with following parameters; 90 kVp, 4-10 mA, 200 µm voxel size. The measurement was performed using Planmeca Romexis 4.6.2.R software (PLANMECA Romexis, Helsinki, Finland). Group differences were compared using the t test or Chi square test where appropriate. In addition to this first analysis, ANOVA (analysis of variance) was conducted in order to compare the right and left sides of the three measurements (ARC; BCB; NCF) with a significance level of 95% (*p* ≥ 0.05) to check whether there were any differences between the sides. All statistical analyses were performed using SPSS 15 (SPSS Inc., Chicago, IL, USA).

## Results

CBCT images of 326 patients (133 male, 193 female) were screened. ACs were identified at total of 113 patients (53 female, 60 male) In our study, the prevalence for male patients was 45,1% and for female patients it was 27,5% (*p*=0,001).

Screened patient group mean age was 43,47±15,27 with ages ranging from 10 to 86 years. The mean age of the female patients with canals was 45,55 ±10,70, and for the male patients it was 47,10±16,16.

While 88 patients had 114 ACs on the right side (53%), 100 ACs were detected on the left side of 80 patients (47%). The distrubition of the canals at left and right sides at patients can be seen in Table 1.

**Table 1:**
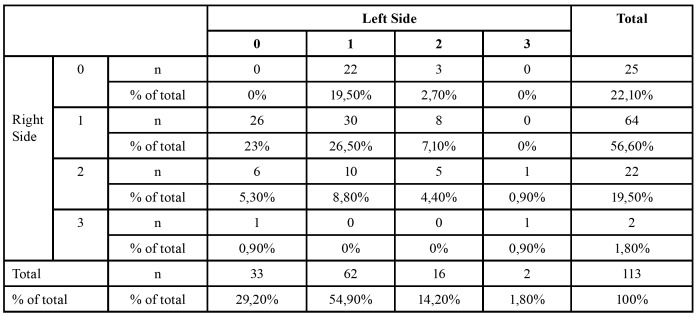
Distribution of patients (with/without AC) regarding to AC number and localization (right/left).

The average diameter of the foramen of the 172 ACs, considered to be a minimum of 0,5 mm, was 1,30 mm (range 0,57-2,88 mm, SD ± 0,44 mm). The number of ACs for which the diameter of the foramen is less than 0,5 mm was 42.

When analyzing the 172 ACs that diameter wider than the 0,5 mm, no significant relationship was found between the age of the patients and the diameter of the ACs (*p*=0,086). No significant relationship was found between foramen diameter and gender (*p*=0,251) and location (*p*=0,869). Median diameters of ACs greater than 0,5 mm in diameter was 1,07 (range 0,53-2,72 mm, SD ± 0,35 mm). Statistically no significant differences were found between median diameter of ACs and age (*p*=0,120) and location (*p*=0,861). But there were significant differences between median diameter and gender. The median diameter of the ACs of the males were found to be significantly higher than females (*p*=0,013).

The ACs were located most frequently at the righ lateral incisor region (n = 36, 17,8%). The lowest number of ACs in anterior maxilla were found between the region of tooth 14 and tooth 15 and between the region of tooth 24 and tooth 25 (Table 2).

Among the age groups 10-19, 20-29, 30-39, 40-49, 50-59, 60-69, 70-79, 80-89 years, the age group with the most ACs was found as 30-39 years and distribution of patients with age groups was shown in Table 3. There was no statistically significant difference between age groups excluding 80-89 (*p*=0,429).

148 ACs (69,15 %) presented a curved communication with the CS. 56 canals (26,16%) had a straight vertical direction from the medial aspect of the pyriform aperture. 10 canals (4,67%) presented a Y-shape branching pattern of the accessory canals within the anterior maxilla (Table 4).

**Table 2:**
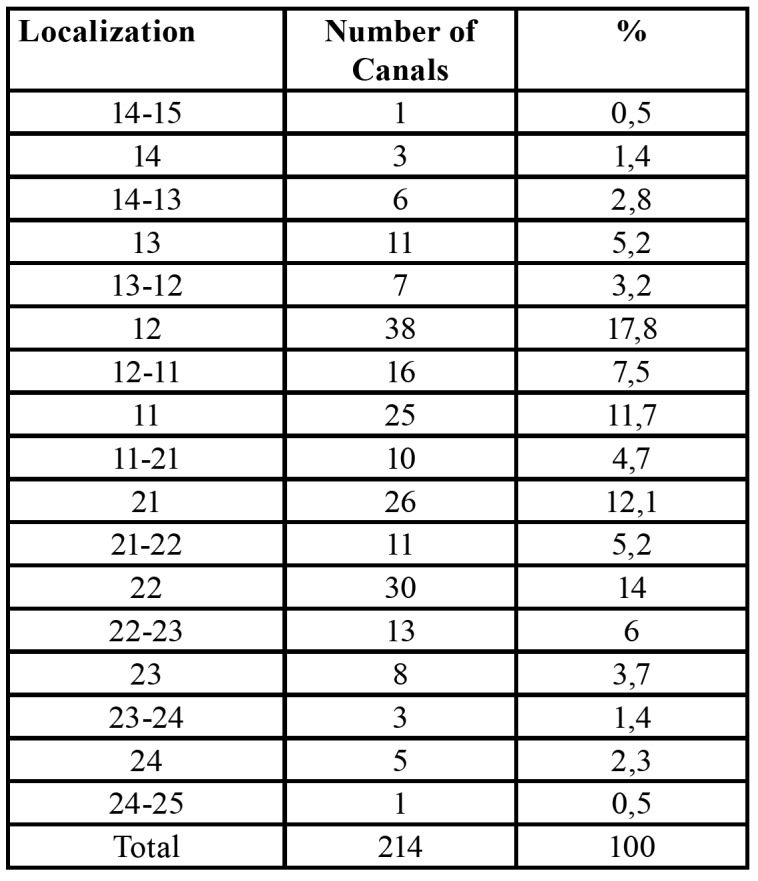
The distribition of the canals at different áreas.

**Table 3:**
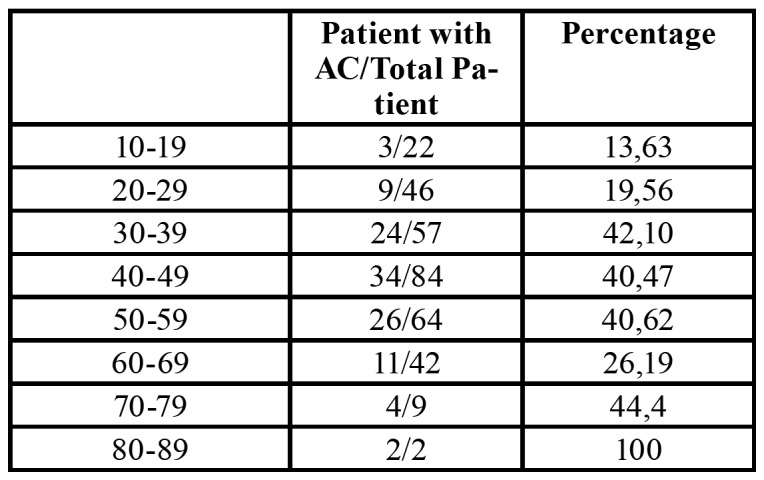
Distribution of patients with age groups.

**Table 4:**
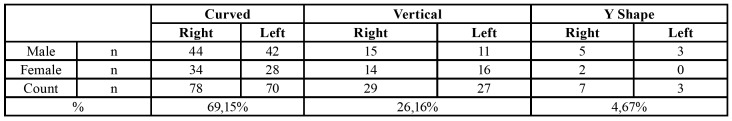
The distribution of the canal shapes at genders and sides.

There was no statistically significant difference for course of the ACs between gender (*p*=0,096).

The mean distance of the AC to the NCF was found 13,83 mm (range 1,61- 26,40 mm, SD±4,10). In female and male groups, this distance was found 13,64 mm and 13,99 mm, respectively. Localization of ACs (*p*=0,819) and gender (*p*=0,548) did not significantly affect this distance. Age (*p*= 0,001) significantly affect this distance. This distance was higher in between the ages of 80-89 (17,32 mm) and then between the ages of 20-29 (15,59 mm).

The mean distance of the AC to the BCB was found 6,60 mm (range 0,80- 12,08 mm, SD±1,82). In female and male groups, this distance was found 6,69 mm and 6,54 mm respectively. Localization of ACs (*p*=0,364) and gender (*p*=0,541) did not significantly affect this distance. But it was found that there was a significant relationship between the age (*p*=0,000) variable and this distance. In this relationship, it was observed that there was an inverse and medium strong relationship. As the age increases, the distance significantly decreases.

The mean distance of the AC to the ARC was found 5,32 mm (range 0 – 17,60 mm, SD±3,19). In female and male groups, this distance was found 5,95 mm and 4,81 mm, respectively. It was found that there was a significant relationship between the gender (*p*=0,009) variable and this distance. This distance was found to be higher in females than males. Age (*p*=0,842 ), localization of ACs (*p*=0,756) did not significantly affect this distance.

Measurement of the mean distance of ACs to the incisive canal in the region of all teeth between # 12 and #22 (including 12 and 22) was found 3,66 mm (range 0- 7,79 mm, SD±1,86). The total number of channels in this group was 156, since the diameter of the 7 canals were less than 0,5 mm and they did not included in the mean value calculation. In female and male groups, this distance was found 3,16 mm and 4,11 mm, respectively. Localization side of ACs (*p*=0,331) did not significantly affect this distance. But it was found that there was a significant relationship between the gender (*p*= 0,002) variable and this distance. This distance was found to be higher in males than females.

A total of 174 channels were found between the lateral teeth located around the incisive canal. The distribution of these canals around the incisive canal is examined, most common locations were right (40,38%), left (37,17%), right-anterior (8,97%), left-anterior (8,33%), right-posterior/left-posterior (1,13%), anterior (1,19%) and posterior (0,64%) respectively.

## Discussion

**Figure 1:**
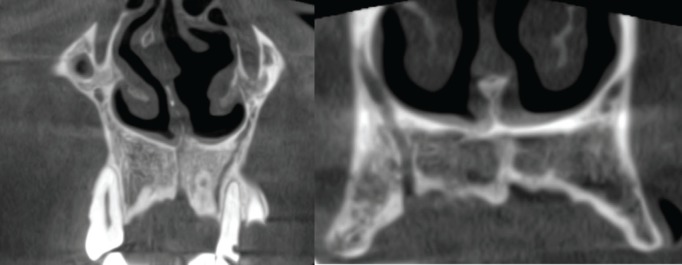
Two examples of canalis sinuosus accessory branch run to the alveolar ridge crest.

The clinicians knowledge about the anatomical variations decrease complication possibility and increase the prognosis (2). Neurovascular bundles in anterior maxilla are important. Due to surgical procedures, they may damage the structures and cause sensory dysfunction (hyperesthesia, paraesthesia or pain) and haemorrhage. Also these bundles may alter the osseointegration and may lead to failure of implants. Lastly, they may mimic a lesion, can cause diagnostic confusion where Shah *et al* reported a case with accessory branch of CS mistakenly prediagnosed as an external root resorption at periapical radiographies (4,10,11) (Fig. 1).

The visualization of the accessory branches of the CS with regular radiographic methods are difficult due to smaller size, the diameter of the accessory canals usually smaller than 1 mm. Also porous cortical layers and variable course suggested as a difficulty factor for diagnosis in conventional radiographic methods (4,10). Machado *et al* report a case who had pain after implant insertion with a close relation to accessory canal and immediate relief after removal of the implant (10).

CS and also the accessory canals are less known variations in clinical practice. Few case reports and studies present them in the literature. With the increasing rate of dental implant procedures and 3D imaging in clinical practice, this variation becomes an attention grabbing variation (12).

Some studies referred CS as a rare variation (13,14). The prevalence of the accessory canals of the CS shows a wide range between 15,7% (11) and 70,8% (12), Oliviera Santos *et al* (11) which reported the lowest prevalence in the literature like Von Arx *et al* (1) include the canals only measured 1 mm or wider. In our study, the prevalance was 34,66%. Of the 326 patients screened, 113 had a total of 214 canals. In our study, every canal which can be detected at images was included in the prevalence not only canals wider than 1 mm like Von Arx (27,8%) (1), Oliviera Santos (15,7%) (11) and Sekerci *et al* (22,3%) (15). This could be the reason for high prevalence according their paper. Ghandourah *et al* (8) reported a prevalence as 67,6% which is higher than our findings, the reason for this might be the used voxel size differences between studies. In our study we used voxel size as 200 µm, Ghandurah *et al* (8) used voxel sizes between 80-250 µm. Significant difference between the prevalences may be derived from variety of reasons like methodological differences (voxel size, using of different CBCT scanners, different exposure parameters, inclusion/exclusion criterias etc), racial differences, study groups’ distribution or may be just coincidental.

Machado *et al* (10) reported a statistically significant difference of AC prevalence where they show male dominance, contrary to Machado, Sekerci *et al* (13) reported a girl’s dominance in paediatric population (10). Von Arx *et al* (1), Oliviera Santos *et al* (11), Ghandourah *et al* (9), Orhan *et al* (12) show no significant difference between genders. In our study in the patients with ACs the male/female ratio was 0,88. This article is in agreement with those previous studies in which gender differences were not statistically significant (*p* = 0.544).

Von Arx *et al* (1) reported increasing frequency of ACs with older ages and reported highest frequency in >60 year older group as 32,9%. But difference in age distribution and number of accessory canals per individual in different age groups was not significantly different. Orhan *et al* reported highest frequency of AC in 50-59 age group (12). Machado reported the highest frequency at 41-60 age group as 55,1%. But they report no significant difference between age groups (10). In our study, 2 patients with in the group of 80-89 age group showed ACs and the prevalence for this group was 100%. Also in 70-79 group (9 patient) the frequency was 44,4% but also the total number of patients was low. As the total number of the patients in these groups was small. We believe these findings are coincidental ones. The most common group other than these groups was 30-39 age group (42,1%) followed by 50-59 age group (40,62%). There was no statistically significant difference between age groups excluding 80-89 group which is in agreement with the literature (*p*=0,429).

No statistically significant difference regarding the frequency of AC and the gender was reported by Oliveira Santos *et al* (11), Von Arx *et al* (1) and Orhan *et al*’s studies (12). Machado *et al* (10) reported significantly higher prevalence in males (58%) than females (46,6%). In our study, the prevalence for male patients was 45,1% and for female patients 27,5% (*p*=0,001). Our findings show male dominance like Machado *et al*’s study (10).

Machado *et al* (10) included the CS which they measured more than 1 mm in diameter such as Oliviera Santos *et al* (11), Von Arx *et al* (1) and Sekerci *et al*’s study (15). In this article, the diameter of the CS evaluation was accepted to be at least 0,5 mm. The average diameter of the AC was 1,19 mm in Machado *et al*’s study (10), which is similar to the average diameter of 1,31 mm reported by Von Arx *et al* (1), 1,2 mm by Sekerci *et al* (15), 1,4 mm in the analysis by Oliveira Santos *et al* (11). In this paper, average palatal/buccal opening of ACs diameter is 1,30 mm and average of ACs median diameter is 1,07 mm. In this study, the diameter of the canal opening and the diameter measured from the middle section of the canal were calculated separately. In average calculated foramens-canals, only canals-foramens wider than 0,5 mm included in the calculation. These values were close to the data obtained in previous studies.

Considering study by Wanzeler *et al* (16) CS’s diameter that is stated that there is a difference between the start and terminal sections. When the literature was searched, it was observed that there was no study other than Wanzeler *et al* (16). In this study, the diameter of CS was measured from where it was opened and the largest in the region and the mean diameter values were calculated in this way. In our study average diameter for canals wider than 0,5 mm median was 1,07 mm and for foramen wider than 0,5 mm was 1,3 mm. Our finding support that the foramen becomes narrower with the upright course.

The distribution of the AC of the literature has shown that there are differences. Ghandourah *et al* have reported to be seen in adults predominantly in region of the central incisors (41.3%) (8). This finding is similar to the study given by Von Arx *et al* (56.7%) (1). In their study, Ghandourah *et al* defined that the area of AC the most commonly seen in adolescents location as the left lateral incisor and canine region (27.8%) (8). This study is parallel to the finding by Sekerci *et al* (15). Manhaes Junior *et al* (9) found that it was next to incisive foramen and Oliveira Santos *et al* (11) reported that it is close to the incisors or canine. Machado *et al* (10) noted that the most frequently seen region is upper maxillary incisors’ palatine. In another study by Orhan *et al* (12), CS was found to be the most common site in maxillary intercentral region. According to this study, CS was mostly found in tooth of 12 (17.8%) followed by the left lateral incisor (%14). Our findings are in agreement with Ghandourah *et al*’s study (8) for the tooth number but not with the side.

In study by Wanzeler *et al* (16), the distance between CS’s terminal portion and the region of alveolar ridge was observed as statistically significant differences associated to the gender. And this distance was found to be higher in males than females. As Oliveira Santos *et al* (11) reported this difference in their studies, it was concluded that males may have differences of size, shape and bone density from females. Since the male’s alveolar ridge size is larger, it is natural that this distance is also concluded. In the study of Manhaes Junior *et al* (9), they measured distance between the CS and ARC, between the CS and BCB and between the CS and NCF in all regions. In female group in this study there was obtained a significant difference in favor of left only distance between CS and BCB measurement. When the results of male group were examined, it was seen that there were no differences between the sides for any of the measurements. In mixed group, there was a difference in favor of left in the distances from the CS to the ARC and to the BCB. In any group, there was no significant difference between the right and left sides in the distances from CS to NCF (9). In our study only significant relationship was seen between the gender (*p*=0,009) and ARC distance. This distance was found to be higher in females than males. These findings, may be originated from males loss tooth earlier than females. As in our study, we did not screen the presence of tooth at relevant locations and no data for the toothless time at relevant area, we can not be sure of this argument. In any group, there was no significant difference between the right and left sides in the distances from CS to NCF, BCB, and ARC.

When the literature was reviewed, it was seen that there was no study measuring the distance between the incisive foramen and ACs. In our study; the distances between the canals located between the teeth 12 and 22 were measured. A total of 156 ACs were found in this region and 2 of these canals were found to open directly to the incisive canal. The foramen diameter of 7 canals was less than 0.5 mm. These 9 canals were not included in the mean intercanal distance calculation. And measurement of the mean distance of ACs to the incisive canal was found 3.66 mm.

In a study by Von Arx *et al*, ACs was classified in three ways according to the course. In 56,7% of canals, it was observed that curved direction the alveolar process. In 41,8% of canals was traced vertical direction from the medial aspect of the pyriform aperture. The remaining 1,5% showed with a Y-shape conFiguration, one branch originating from the canalis sinuosus and the other branch coming from the medial aspect of the nasal floor (1). In our study; curved prevalence is found as 69,15%. The prevalence of vertical tracing is 26,16% and Y-shape conFiguration of CS incidence is 4,67%. Not the percentages but the frequency aligment is in accordance with the Von Arx *et al*’s (1) study.

Study by Machado *et al* (10) the most common ends of the AC trajectories were located palatal to the anterior maxillary teeth (91.1%; 887/974), and less frequently in a buccal position (5.1%; 50/974) and in a transversal position (3.8%; 37/974). In our study only 3 of the cases show buccal opening of the ACs.

The present study provides new information to the literature concerning the identification of the localization of ACs with the incisive foramen.

In conclusion CS is an anatomical structure between the alveolar ridges on the inferior wall of the nasal cavity. It is known that complications such as nerve damage, haemorrhage, unexpected bleeding as a result of damage of ASA nerves and branches in this channel are known. So it is important that in the surgical procedures of the anterior maxillary region, identification of CS with the possible accesory branches’ anatomy with the CBCT helps avoid injuries and malpractices. The aim of this study is to determine the morphology and location of the ACs and make correlations with gender, age, and distance of this canal to important adjacent structures on the region and contribute to the literature.
